# Non-Invasive PPG-Based System for Continuous Heart Rate Monitoring of Incubated Avian Embryo

**DOI:** 10.3390/s20164560

**Published:** 2020-08-14

**Authors:** Ali Youssef, Daniel Berckmans, Tomas Norton

**Affiliations:** Faculty of Bioscience Engineering, Katholieke Universiteit Leuven (KU LEUVEN), Kasteelpark Arenberg 30, 3001 Heverlee/Leuven, Belgium; ali.youssef@kuleuven.be (A.Y.); daniel.berckmans@kuleuven.be (D.B.)

**Keywords:** embryonic heart rate, photoplethysmography (PPG), continuous wavelet transform (CWT), spectral entropy

## Abstract

The chicken embryo is a widely used experimental animal model in many studies, including in the field of developmental biology, of the physiological responses and adaptation to altered environments, and for cancer and neurobiology research. The embryonic heart rate is an important physiological variable used as an index reflecting the embryo’s natural activity and is considered one of the most difficult parameters to measure. An acceptable measurement technique of embryonic heart rate should provide a reliable cardiac signal quality while maintaining adequate gas exchange through the eggshell during the incubation and embryonic developmental period. In this paper, we present a detailed design and methodology for a non-invasive photoplethysmography (PPG)-based prototype (Egg-PPG) for real-time and continuous monitoring of embryonic heart rate during incubation. An automatic embryonic cardiac wave detection algorithm, based on normalised spectral entropy, is described. The developed algorithm successfully estimated the embryonic heart rate with 98.7% accuracy. We believe that the system presented in this paper is a promising solution for non-invasive, real-time monitoring of the embryonic cardiac signal. The proposed system can be used in both experimental studies (e.g., developmental embryology and cardiovascular research) and in industrial incubation applications.

## 1. Introduction

The chicken embryo is not only an important product in the global demand for chicken meat but is also a widely used experimental animal model in developmental biology research, the study of physiological responses and adaptation to altered environments, and neurobiology research [1,2,3]. Additionally, embryonic chorioallantoic membrane (CAM) assays have been widely used to study angiogenesis and tumour cell invasion metastasis. The CAM-based assay has many advantages, such as its highly vascularised nature, which promotes the efficiency of tumour cell grafting, and its simplicity and cost effectiveness [4]. Moreover, chicken eggs can be incubated to any developmental stage, simplifying experimental design. Furthermore, within two to three days of incubation, chick embryos gastrulate, neurulate, and fold into three-dimensional (3D) animal models with beating hearts and complex nervous systems [5]. Many parameters of interest can be monitored during the development of the embryo. The heart rate (HR) is an important physiological variable useful as an index reflecting the embryo’s natural activity and traditionally has been one of the most difficult parameters to measure [6].

The heart is the first organ to function during the developmental stage of the avian embryo [7]. Maturing tissues and organs require a higher oxygen consumption during later stages of development. This can only be met by an increasing oxygen delivery when blood flow finally takes place. The heart rate of a chicken embryo is therefore an important autonomic controlled index of the cardiovascular system [8]. Additionally, the embryonic heart rate (HR) during incubation and before hatching can be a useful physiological parameter for studying the regulation mechanisms of the cardiovascular system and the thermoregulatory development. Photoplethysmography (PPG) is a measurement of the heartbeat rate based on changes in the rate of blood flow. Unlike an electrocardiogram (ECG), which measures the heart rate by placing electrodes on the patient’s chest to measure electrical potential, PPG is a low cost, simple, non-invasive optical measurement based on the reflection of light and is used in the biomedical field to detect blood volume changes in the microvascular bed of tissue through red and infrared lights [9].

In the literature, cardiogenic signals of avian embryos are detected using various sensors noninvasively, semi-invasively, or invasively while maintaining adequate gas exchange through the eggshell [10,11].

As cited by Bellville [12] and Romanoff [13], the early research of Bogue [14] in 1932 showed that the heart rate of chicken embryos could, at that time, only be studied using an electrocardiogram (ECG). In this technique, at least three electrodes have to be inserted into the tissues surrounding the embryo.

Later, Bamelis et al. [11] used an ECG measuring technique in which the electrodes were positioned between the shell and the outer membrane. However, due to the exposed holes in the eggshell the gaseous exchange (i.e., water vapour, oxygen and carbon dioxide) between the egg and it surrounding environment will change dramatically [11]. Moreover, long-term recording of the heart-rate in this way involves risks due to the increased possibility of bacterial infections passing through the exposed holes.

The same concerns also exist in the case of the impedance cardiogram (ICG), developed by Tazawa and Whittow [15], and the pulse-oximetry technique first presented by Lewin et al. [8].

To overcome these concerns, different researchers have developed various techniques to measure the heart rate. Several invasive and less-invasive techniques have been tested and demonstrated to measure the longer-term heart rate and heart rate variability of the chicken embryo [16,17,18].

The ballistocardiogram (BCG) is based on slight movements or vibrations of the eggshell, caused by contraction of the embryonic heart, which can be detected by piezoelectric sensors [19] or optically via laser interference [3]. The acoustocardiogram (ACG) uses the effect of the pulsatile air movement across the eggshell, detected by microphone [20] or differential pressure transducer [21]. In their work [20], Rahn et al. developed a technique that involved placing an egg in a tightly sealed vessel containing a condenser microphone. Akiyama et al. [10] measured the instantaneous heart rate (IHR) in chicken embryos using an acoustocardiogram (ACG) from day 12 until hatching. They found that the IHR comprised transient bradycardia and tachycardia, which first developed on day 14 and 16 in most embryos, respectively.

In their study [8], Lewin et al. introduced a PPG-based technique to measure the chicken embryonic heart rate. Youssef et al. [22] introduced a novel approach for the real-time, semi-invasive measurement of the embryonic heart rate based on image photoplethysmographic (iPPG) techniques via a small window (1 cm^2^) in the eggshell. Although the technique developed by Youssef et al. [22] provided continuous and real-time measurements of the developing embryo during incubation, it is an invasive technique that requires careful handling to prevent contamination from taking place. Yu et al. [23] used the PPG technology to non-invasively distinguish between live and dead chicken embryos. In a recent study [24], Phuphanin et al. developed a smartphone-based algorithm for non-invasive detection of the embryonic heart rate.

Although a substantial amount of research has been conducted on continuous long-term monitoring of the HR in chicken embryos, to date, no practical and affordable technique, implementable in commercial incubators, is available. In the present work, we present a full description of a prototype of a non-invasive PPG-based system, including hardware and a real-time algorithm, for continuous long-term heart rate monitoring of the developing chicken embryo during incubation.

## 2. Photoplethysmographic (PPG)-Based Embryonic Heart Rate Extraction

In general, the photoplethysmographic (known most commonly as PPG) system consists of a light source (usually a light-emitting diode) and a photodiode to detect the light. When a light-emitting diode (LED) emits light, it passes through living body (tissue) and can be absorbed by the surrounding substances such as arterial blood, venous blood, bone, and skin [25]. The absorption of this light can either be constant (bone and skin) or alternating (arterial blood). The alternating occurrence in arterial blood is attributable to the blood flow rate or volume per unit of time, which is greater during the systolic phase than during the diastolic phase. Depending on the relative position of the photodiode to the light source, two different modes of operation exist, namely, transmittance and reflective modes. In the reflectance mode, the light emitter is placed next to the detector, which measures the backscattered or reflected light from the tissue (body). Alternatively, in the transmittance mode, the photodiode is placed opposite the light emitter in such a manner that the photodiode only detects the transmitted light through the body.

Many studies have previously indicated that green light (495–570 nm) shows a higher absorptivity for oxygenated blood than other spectra (e.g., [25,26]). In general, short wavelengths (i.e., green and blue) are known to have a lower penetration depth (δ) than longer wavelengths (i.e., red and infrared) [27,28]. This is defined as the depth when the light intensity decreases to 37% of the intensity on the incident surface [26]. Therefore, short wavelengths are more common in detecting superficial blood flow while deeper tissue movements have less impact.

The penetration depth is described as [28]:

(1) $$\delta =\frac{1}{\sqrt{3\mu_{a}\left(\lambda \right)\cdot \left(\mu_{a}\left(\lambda \right)+\mu_{s}\left(\lambda \right)\right)}}$$

where δ is the penetration depth (mm), and $\mu_{a}$ and $\mu_{s}$ are the absorption and scattering coefficients (cm^−1^), respectively. The penetration depth depends mainly on the extinction coefficient of the tissue or the material through which the light passes. This extinction coefficient is defined as the sum the absorption and scatter coefficients (i.e., $\left(\mu_{a}\left(\lambda \right)+\mu_{s}\left(\lambda \right)\right)$). As known fact, light travels further through tissue with low absorptive and scatter properties [26]. Because wavelengths such as red and infrared have low coefficients in most biological tissue, these wavelengths are used in the transmittance mode.

### 2.1. Hardware Design and Prototype (Egg-PPG)

**Figure 1:**
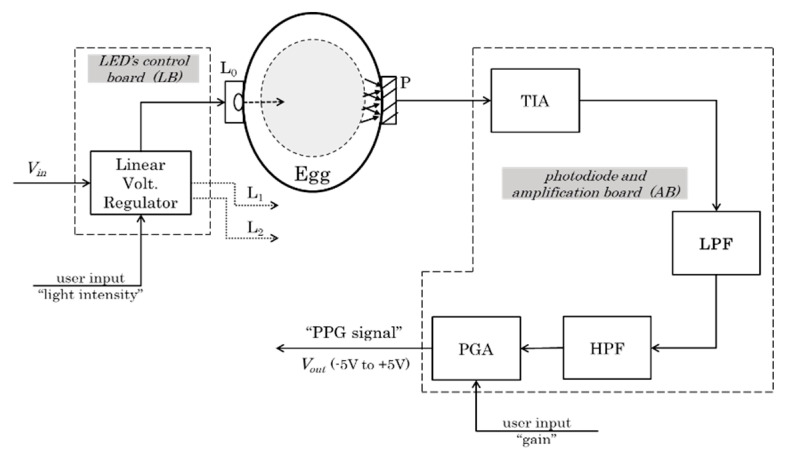
Main hardware components of the developed prototype (Egg-PPG) shows the LED control circuit board (**LB**) for the infrared LEDs (**L_0_**, **L_1_**, and **L_2_**), photodiode (**P**), and the photodiode and amplification circuit board (**AB**) includes a transimpedance amplifier (**TIA**), low-pass filter (**LPF**), high-pass filter (**HPF**), and programmable gain amplifier (**PGA**).

**Figure 2:**
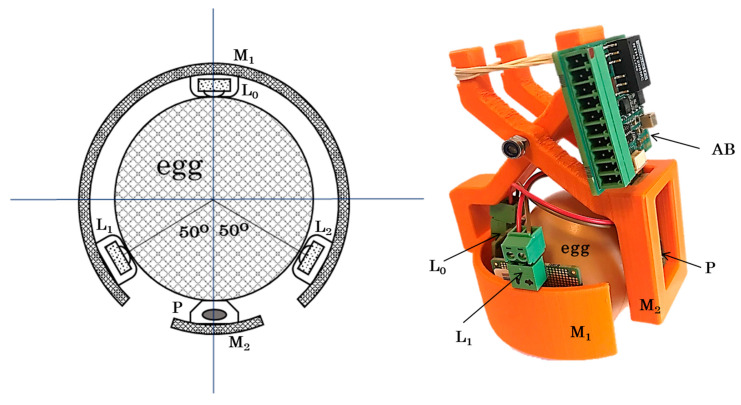
(left graph) a top-view schematic representation of the developed Egg-PPG prototype shows the relative positions of the three infrared LEDs (**L_0_**, **L_1_** and **L_2_**), photodiode (**P**) and the two sections **(M_1_** and **M_2_**) of the Egg-PPG housing system (**HS**), (right graph) the corresponding photographic picture of the Egg-PPG prototype shows the photodiode and amplification circuit board (**AB**).

The main components of the designed and developed Egg-PPG prototype are presented as a block diagram in Figure 1. The Egg-PPG prototype (Figure 2) consists of the two main components, namely, the light source system and the photodiode system.

#### 2.1.1. Light Source System

The light source system comprises the following:

(a) The light emitting diodes (LEDs)

Three infrared LEDs (from Würth Elektronik, Niedernhall, Germany), L_0_, L_1_, and L_2_ (Figure 1) with emitting peak wavelength ($\lambda_{peak}$) of 945 nm and maximum radiant intensity of 300 mW·Sr^−1^ (at ~1 A) are used as the light source in the Egg-PPG.

(b) The LED control board

Due to the continuous changes in the internal physical and optical properties of the incubated egg resulting from embryonic development, the Egg-PPG was designed to adapt the light intensity of the three LEDs to ensure a high-quality PPG signal during the embryonic developmental stages. Thus, the LED control circuit board (LB) was developed to control the light intensity of the LEDs via a linear voltage regulator (LVR). The developed LED control board is provided with an external input terminal (0–5 DC-Voltage), which allows the user to externally control the LED’s current and consequently regulate the LED’s intensity between 0 and 300 mW·Sr^−1^.

#### 2.1.2. Photodiode System

The Photodiode system comprises the following:

(a) Photodiode light sensor

One photodiode (SFH 2201 OSRAM), P (Figure 1), characterised with a wavelength of maximum sensitivity of 950 nm and radiant sensitivity area of 8.12 mm^2^, is used in the Egg-PPG.

(b) Amplification board

When reflected/transmitted light is partly absorbed by the photodiode, a very small current $I_{P}$, with an order of nano-amperes (photodiode maximum current = 74 nA), is produced. This photodiode maximum current ($I_{Pmax}$ = 74 nA) is equivalent to the direct current (DC) signal of the received light. The embryonic cardiac waves appear as a small alternative current (AC) signal that is superimposed on the baseline DC signal. Hence, an amplification circuit board (AB see Figure 2) was developed to amplify and condition the photodiode signal (Figure 1). The output of the amplification board is a PPG voltage signal ($V_{out}$ = −5 V to +5 V), which can be read using a suitable data acquisition interface. The developed AB consists of the following main components:

- Transimpedance amplifier

The transimpedance amplifier (TIA see Figure 2) converts the very small current $I_{P}$, produced by the photodiode, into a readable voltage. The TIA has a gain stage equal to the feedback resistor value $R_{feedback}$, where the voltage output of the TIA is given by $V_{TIA}=I_{P}\times R_{feedback}$.

- Analog filters

The amplification circuit board contains a low-pass filter (LPF) with a cut-off frequency of 16 Hz to filter out the high frequency TIA noise, which originate mainly from the input voltage noise (noise gain), the input current noise, and the thermal noise (resistor). Additionally, a high-pass filter (HPF), with a cut-off frequency of 23 mHz eliminates the DC-offset originating from the constant LED light.

- Programmable gain amplifier

A programmable gain amplifier (PGA) provides control of the TIA gain with 10 gain levels from −10 to −100 dB (in 10 dB steps) via an external digital I/O interface. The PGA provides the possibility and flexibility to adapt the received PPG signal to maximize the signal-to-noise ratio (SNR).

### 2.2. Embrypnic Cardiac Wave Extraction Algorithm and Heart Rate Calculation

**Figure 3:**
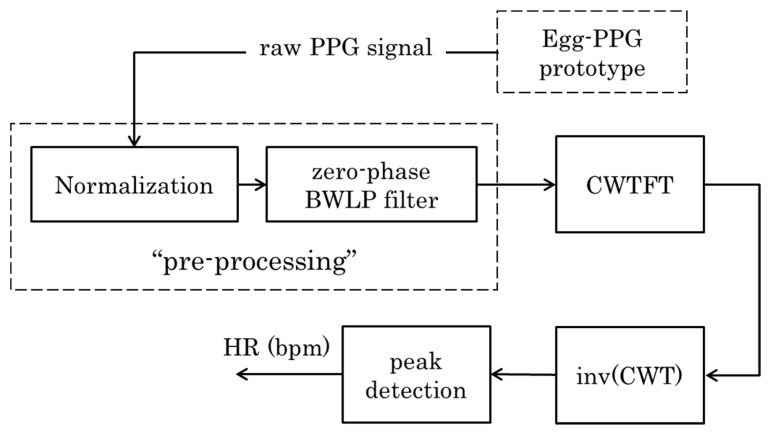
Block diagram showing the main signal processing steps to extract the embryonic heart rate using the Egg-PPG prototype. The block diagram includes the pre-processing, continuous wavelet transform Fourier transform (**CWTFT**) algorithm, inverse continuous wavelet transform (**invCWT**), and peak detection algorithm blocks.

The main components of the continuous wavelet transform (CWT)-based embryonic cardiac wave (ECW) extraction algorithm are depicted in the following block diagram (Figure 3).

#### 2.2.1. Pre-Processing of PPG Signals

**Figure 4:**
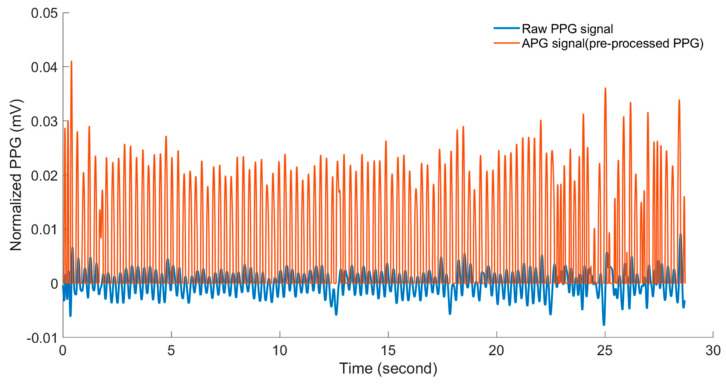
The acceleration plethysmogram (APG) signal (red line) resulting from pre-processing compared to the raw PPG signal (blue line), obtained from an incubated fertile egg at embryonic day ED09.

Firstly, the PPG signals are normalized to zero mean and unit variance [29]. The normalized signals are filtered using a zero-phase 4th order Butterworth low pass (BWLP) filter with a cut-off frequency of 7 Hz. This cut-off frequency is chosen to contain, in the filter passband, the documented (e.g., [3,10,14,30]) physiological heart rate range (160–300 bpm) of the chicken embryo during different developmental stages. The Butterworth filter provides a maximally flat passband and the zero-phase implementation to preserve the embryonic cardiac wave. The second derivative of the PPG signal, also called the acceleration plethysmogram (APG), shows more defined peaks than those of PPG signal, which more accurately detect the heart rate [31]; the APG was used in the presented approach. Figure 4 shows an example of the resulting APG signal after pre-processing compared with the raw PPG signal obtained from an incubated fertile egg at embryonic day ED09. To further distinguish the cardiac wave peaks, positive values of the APG signal are squared, whereas negative values are equated to zero (see an example in Figure 4).

#### 2.2.2. Wavelet Analysis and Peak Detection

Wavelet transform (WT) is a spectral estimation technique performed by breaking a general function into an infinite series of wavelets [29]. In the biomedical engineering field, wavelet transform (WT) is often preferred over fast Fourier transform (FFT) in signal processing because most of the physiological signals are non-stationary in nature, which makes WT a viable method. The wavelet transform can be used to analyse time series that contain nonstationary power at many different frequencies [32,33]. Using WT, the signals in the time domain are mapped into the frequency domain to preserve both the time and frequency information.

- Continuous Wavelet Transform method

Generally, in the continuous wavelet transform (CWT) method, a specific wavelet centred about a given frequency is computed from the mother wavelet by scaling and shifting it. In this manner, the length of the wavelet contains the same number of centre (also called peak) frequency cycles. For a scale parameter *s* > 0, and a position parameter *b*, which defines a translation of the wavelet and indicates the time localization, the CWT can be given by [33]:

(2) $$C\left(s,b\right)=\int_{-\infty }^{+\infty }x\left(t\right)\frac{1}{\sqrt{s}}\psi^{*}\left(\frac{t-b}{s}\right)dt$$

Wavelet analysis is performed by convoluting the signal under investigation (i.e., the APG signal), x(t), with a mother wavelet, ψ(t) $\psi^{*}\left(t\right)$ is the complex conjugate of the analysed mother wavelet, and the term $\frac{1}{\sqrt{s}}$ is an energy normalized factor (the energy of the wavelet must be the same for different s values of the scale). As the scale s increases, the wavelet is compressed, its spectrum dilates, and the peak frequency shifts to a higher value. Conversely, when s decreases, the wavelet dilates, its spectrum is compressed, and the peak frequency shifts to a lower value [34]. In practice, the CWT is usually computed over discrete values of the s scale in the range of continuous values. To approximate the continuous wavelet transform, the Equation (2) should be applied N times for each scale, where N is the number of points in the discrete signal f(t) [33]. In general, the classic CWT is time consuming and the computing power required is too high for it to be applied in real-time. Hence, more efficient algorithms have been developed to reduce the required computational power and time of CWT calculation (e.g., [35,36,37]).

In the presented approach, the CWT is calculated using fast Fourier transform (CWFT) [37] that allows N convolutions to be computed simultaneously, and is thus more suitable for real-time applications. The CWFT algorithm implements the following steps:

- Compute the discrete Fourier transform (DFT) of the analysed signal x(n), including N samples, using Fast Fourier Transform (FFT) as follows:
  (3) $$\hat{x}\left(k\right)=\sum_{n=0}^{N-1}x\left(n\right)e^{-i\frac{2\pi }{N}nk},\;k=0,1,2\cdots N-1$$
  where k is an index of frequency.
- Obtain the DFT ($\hat{\psi }\;$) of the analysed wavelet (ψ) at the appropriate angular frequencies as follows:
  (4) $$\hat{\psi }\left(k\right)=\sum_{n=0}^{N-1}\psi \left(n\right)e^{-i\frac{2\pi }{N}nk},\;k=0,1,2\cdots N-1$$
- Scale the DFT of the analysed wavelet at different scales to ensure different scales are directly comparable.
  To obtain the unit energy for each scale s, the wavelet function is normalized using the following formula:
  (5) $$\hat{\psi }\left(s\omega_{k}\right)=\sqrt{\frac{2\pi s}{\Delta t}}\hat{\psi }\left(s\omega_{k}\right),$$
  where $\Delta t=1/f_{s}$ is the sampling period with $f_{s}$ is the sampling frequency and $\omega_{k}=\frac{2\pi k}{N\Delta t}$.
- Compute the product of the signal DFT and the wavelet DFT over all of the scales. Invert the DFT to obtain the CWT coefficients as follows:
  (6) $$W_{s}\left(b\right)=\frac{1}{N}\sqrt{\frac{2\pi s}{\Delta t}}\sum_{k=0}^{N-1}\hat{x}\left(\frac{2\pi }{N\Delta t}k\right){\hat{\psi }}^{*}\left(s\frac{2\pi }{N\Delta t}k\right)e^{i\frac{2\pi }{N}kb}.$$

The Gaussian function is perfectly local in both time and frequency domains and is indefinitely derivable; a derivative of any order (m) of the Gaussian function may be a Wavelet Transform (WT). A typical cardiac pulse event in the PPG signal consists of two modulus maxima with different signs of $W_{s}\left(s,b\right)$ (i.e., maxima and minima) [38]. Sahambi et al. [38] used a first-order odd function to detect the QRS complex in the ECG signal.

Hence, a first-order derivative of Gaussian (DOG) wavelet was chosen for the presented work because, in general, the obtained scalograms (scales “s” vs. positions “b”) using this wavelet showed clear frequency contents within the expected pulse rate ranges of the chicken embryo. The first-order DOG wavelet is given as follows:

(7) $$\psi \left(t\right)=-t\;exp^{\left(-\frac{t^{2}}{2}\right)}.$$

#### 2.2.3. Power Spectral Entropy and Embryonic Cardiac Wave Recognition

Spectral entropy is used in different applications as a entropy-like index to identify the relative disorder in time dynamics of biological, ecological, and physical systems [39,40]. Detection of the specific waveform of the embryonic cardiac wave (ECW) is a challenging process, particularly in the presence of non-stationary noise, such as motion artefacts, and during the early stages of embryonic development due to the relatively weak cardiac signal. To overcome these problems in applications such as speech recognition and end-point detection, the entropy-based algorithm has been proposed by some investigators (e.g., [39,41]). In the present work, we used an entropy-based algorithm for automatic ECW detection. The application of the entropy concept for ECW recognition is based on the assumption that the spectrum of the PPG signal is more organised (lower entropy) during the segments of cardiac events than during noise segments. The power spectral entropy ($E_{S})$ of the PPG signal is computed as follows [39]:

- The probability density function (PDF) of the spectrum of the PPG signal can be estimated by normalisation over all of the frequency components:
  (8) $$P_{i}=\frac{S\left(f_{i}\right)}{\sum_{k=1}^{N_{f}}S\left(f_{k}\right)},\;i=1,2,\cdots N_{f}$$
  where $P_{i}$ is the probability density for the $S\left(f_{i}\right)$, which is the spectral energy of the ith frequency component $f_{i}$, obtained by fast Fourier transform (FFT) and $N_{f}$ is the total number of frequency components in the FFT.
- Then, the spectral entropy ($H_{n}$) of the PPG nth segment is calculated as follows:
  (9) $$H_{n}=-\overset{N_{f}}{\underset{i=1}{\sum }}P_{i}\log_{2}P_{i}$$

Usually, the spectral entropy (H) is normalized by dividing Equation (8) by $\log_{2}N_{f}$, which represents the maximal spectral entropy of white noise, uniformly distributed in the frequency domain. In this paper, we propose a different approach of normalization of the spectral entropy, namely, by dividing the PPG spectral entropy ($H_{n}$), Equation (8), by the spectral entropy of a PPG segment obtained from an infertile egg ($H_{\infty }$). The normalized spectral entropy ($E_{S}$) is given as follows:

(10) $$E_{S}=\frac{H_{n}}{H_{\infty }}=\frac{\sum_{i=1}^{N_{f}}P_{i}\log_{2}P_{i}}{\sum_{i=1}^{N_{f}}P_{\infty i}\log_{2}P_{\infty i}},$$

where $P_{\infty }$ is the probability density of the PPG segment obtained from an interfile egg, which represents, in our case, the maximal spectral entropy. Hence, it is expected that the normalized spectral entropy ($E_{S}$) of the PPG signal is equal to or closer to unity in the absence of cardiac events.

#### 2.2.4. Peak Detection and Heart Rate Calculation

In general, the heartbeat can be estimated by calculating the time between the peak intervals in the PPG signal. The peak is detected by calculating the local maxima of the decoupled cardiac pulse signal X(n) within a predefined interval (window) I and finding $n_{o}\in I$ such that:

(11) $$X\left(n_{o}\right)\geq X\left(n\right),\;\forall n\in I$$

The algorithm then repeats the procedure of the tallest peak and iterates until all peaks are considered.

## 3. Experiment and Measurements

### 3.1. Incubation and Incbuated Eggs

Thirty fertile eggs of broiler chickens (breed Ross 308 and flock age of 42 weeks) were incubated in an experimental incubator (a detailed description of the experimental incubator can be found in [42]) at 37.8 °C, with relative air humidity of 60%, and automated turning every two hours. Four infertile eggs were also placed as a control (blank) test inside the incubator during the whole incubation period.

### 3.2. Data Acquesition and PPG Measurments

A three-dimensional (3D) printed plastic housing system (HS in Figure 2) was designed to contain the three LEDs (L_0_, L_1_, and L_2_) and the photodiode (P) around the egg (see Figure 2). The housing system (HS) consists of two sections: the largest (M_1_) was designed to hold the three LEDs around the egg with the possibility of placing each LED around the vertical axis of the egg (see Figure 2); the small section (M_2_) held the photodiode (P) tightly around the egg.

The PPG signal obtained from the Egg-PPG prototype was read using a National Instrument^®^ USB-6009 multifunction data-acquisition (DAQ) device as an interface between the Egg-PPG and the computer. A customized MATLAB script was developed to interface/communicate with the Egg-PPG prototype through the National Instrument^®^ USB-6009. Using the developed MATLAB interface script, the PPG signal was acquired at a sampling rate of 128 Hz, and the interface controlled both the light intensity of the three LEDs and amplification gain of the acquired PPG signal.

### 3.3. Detection of Embryonic Cardiac Wave

During the first 24–30 h of incubation, blood and vascular development initiate both in the form of extra-embryonic blood islands and intra-embryonic endothelial cell specification and differentiation [43,44]. The first major blood vessels of the embryo, the paired dorsal aorta, are formed at 30–35 h of development [44,45,46], and a beating heart is apparent by 38–42 h of development [43,47]. Thus, theoretically, the embryonic cardiac wave (ECW) is detectable between the second and third days of incubation. However, this is not the case in reality due to many practical difficulties related to the optical properties of the incubated eggs and the weak cardiac signal, especially in such an early stage of development.

Successful detection of the ECW using the Egg-PPG prototype is dependent on the choice of suitable LED intensity (I) and amplification gain (G). In this phase of the investigation, starting from embryonic day ED03, a manual search for the ECW was performed by changing LED intensity and amplification gain. In total, combinations included eight intensity levels (from 50 to 300 mW·Sr^−1^ in 50 mW·Sr^−1^ dB steps) levels and 10 levels of gain (from −10 to −100 dB in 10 dB steps) to search for the ECW. For each combination, a segment of 60 s of PPG measurement was recorded. After performing the pre-processing step, spectral analysis was performed on the recorded PPG segment. Together with the visual inspection, the spectral analysis results were used to detect the presence of the ECW.

Once the ECW was detected, the PPG signal was acquired daily from three different fertile eggs. Each day the PPG measurement was performed for 10 min per egg. The daily selection of the test eggs was performed by randomly picking eggs from the incubation tray.

## 4. Results and Discussion

### 4.1. Detection of Embyonic Cardiac Wave and Signal Quality

The manual search for the embryonic cardiac wave (ECW) showed that it is only possible to detect an ECW starting from embryonic day ED07 using the Egg-PPG prototype. The results showed that the ECW is detectable from ED07 until ED19 using a light intensity range within I = 150 to 300 mW·Sr^−1^ and amplification gain range within G = −50 to −100 dB.

The daily acquired PPG signals within ED07 until ED18, from all tested eggs, were divided into segments of 10 s each (i.e., 1280 data samples) to be processed individually. In total, 180 PPG segments were analysed per each embryonic day (ED) from ED7 until ED18. After the pre-processing step (see Section 2.2.1), the continuous wavelet transform of each segment was computed using the CWTFT algorithm. The challenging step of embryonic cardiac wave (ECW) decoupling using CWT is the selection of the most suitable set of scales (s).

**Figure 5:**
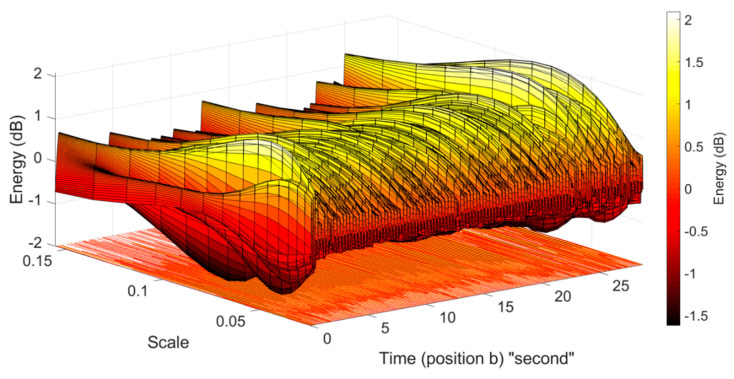
The scalogram shows the distribution of the calculated energy using the CWTFT algorithm for the PPG segment, obtained from an incubated fertile egg at embryonic day ED09; most of the energy dominated the scale range from 0.05 to 0.10.

The suitable set of scales (s) should contain most of the energy of the ECW. We found that the energy of the cardiogenic pulse signals, in all acquired PPG segments, was dominated by five scales between 0.05 and 0.10. Figure 5 shows an example scalogram displaying the distribution of the calculated CWT coefficients (and energy) over the different scales (s), based on the PPG signal obtained from fertile eggs at embryonic ED09. The resulting scalogram (for example, Figure 5) showed that most of the energy (calculated from CWT coefficients) dominated the selected scale range (0.05–0.10).

### 4.2. Embryonic Cadiac Wave Extraction and Heart Rate Calculation

**Figure 6:**
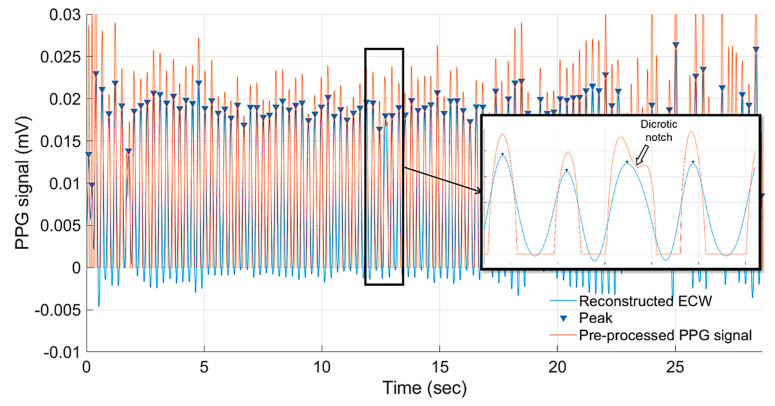
Detected cardiac peaks based on the reconstructed embryonic cardiac wave (ECW) in comparison to a pre-processed PPG segment obtained from an incubated fertile egg at embryonic day ED09. The magnified graph shows that the developed ECW extraction algorithm is not susceptible to false readings of the dicrotic notch.

The developed CWT-based ECW extraction algorithm was used to extract and reconstruct the ECW from each PPG segment, obtained from the tested fertile eggs, during the incubation period between ED07 until ED18. The instantaneous embryonic heart rate (bpm) based on the interpeak interval was calculated using the peak detection algorithm. The results showed that the reconstructed ECW in all cases was not affected by the dicrotic notch, which is a secondary upstroke in the descending part of a pulse tracing corresponding to the transient increase in aortic pressure upon closure of the aortic valve [48], which can lead to a false reading of the peaks (Figure 6).

**Figure 7:**
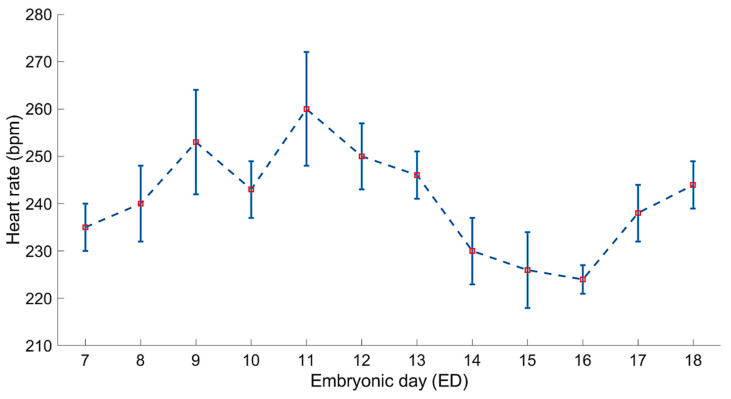
Average ± standard deviation (error bars) of estimated embryonic daily heart rate based on the acquired PPG segments using the developed Egg-PPG prototype and embryonic cardiac wave extraction algorithm.

In Figure 7, the daily averages and standard deviations (represented as error bars) of the estimated embryonic heart rate are shown as calculated from the acquired daily PPG segments between embryonic days ED07 and ED18. The resulting estimations of the daily embryonic heart rate, using the developed Egg-PPG and ECW estimation algorithm, are comparable to the documented (e.g., [3,10,14,22]) heart rates using different techniques (e.g., ECG, ICG, Pulse-Oximetry, and semi-invasive iPPG-based system).

Although the estimated embryonic heart rate was within the expected and documented daily embryonic heart rate, the developed Egg-PPG system together with the ECW extraction algorithm had not yet been validated. A validation technique based on visual inspection (labelling) of the ECW and manual counting of the peaks was used as a ground-truth heart rate (reference). In total, 30 PPG-segments were visually labelled, and HRs were manually calculated and then compared to the corresponding estimated HR using the developed ECW extraction algorithm. Results showed that the average estimated HRs using the developed ECW extraction algorithm were in agreement with the manually calculated HRs with an accuracy of 98.7%. An ECG-based heart rate measurement technique, as reported by Aubert et al. [1], was attempted as a gold standard for this work. However, we did not succeed in obtaining a reliable measurement of the embryonic ECG.

### 4.3. Real-Time Heart Rate Monitoring Algorithm

**Figure 8:**
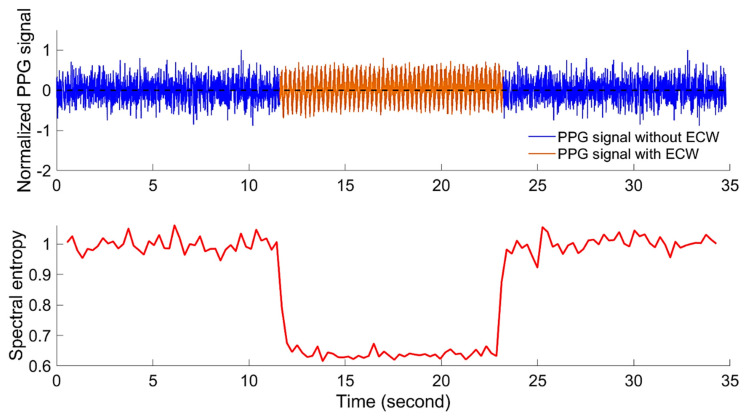
Normalized PPG signal with and without the embryonic cardiac wave (ECW) and the corresponding normalised spectral entropy ($E_{S}$).

A significant challenge to realizing a robust real-time embryonic heart rate monitoring system is developing an automatic ECW detection algorithm. As mentioned earlier, the reliability of the acquired PPG signal is dependent on the selected LED intensity I and amplification gain G. Therefore, to find the most suitable I and G at which the ECW is detectable, a search over all possible combinations of both variables is needed. Additionally, a reliable feature variable is needed as an indicator for the ECW. In this study, normalised spectral entropy ($E_{S}$) was used as a feature variable to detect the ECW. The assumption is that the PPG epoch with ECW contains more information (low spectral entropy) in comparison with the PPG epoch with no ECW, as is the case in an infertile egg. To test this approach, the normalised spectral entropy of 25 PPG segments were acquired from infertile eggs (i.e., containing no ECW) and a further 25 segments were acquired from fertile eggs with a labelled ECW. The $E_{S}$ values of the PPG segments that contained no ECW were found to always be around 1 ± 0.04, whereas the PPG segments with labelled ECW epochs showed lower levels of $E_{S}$ (0.65 ± 0.1). Figure 8 shows that a PPG segment (upper graph) consists of two epochs of PPG signal with no ECW, sandwiching another epoch with the labelled ECW and the corresponding normalised spectral entropy (lower graph). As shown in Figure 8, the plot of $E_{S}$ differentiates the epoch with the ECW from that without.

Based on the 95% percentile of all the ECW epochs, a spectral entropy ($E_{S}$) threshold confidence interval between 0.56 (lower threshold) and 0.69 (upper threshold) in which 95% of the ECW epochs exist was defined. The estimated thresholds were calculated based on the defined PPG signal sampling rate (128 Hz) and the experimental conditions. Therefore, further sensitivity analysis and validation experiments are planned in future studies.

**Figure 9:**
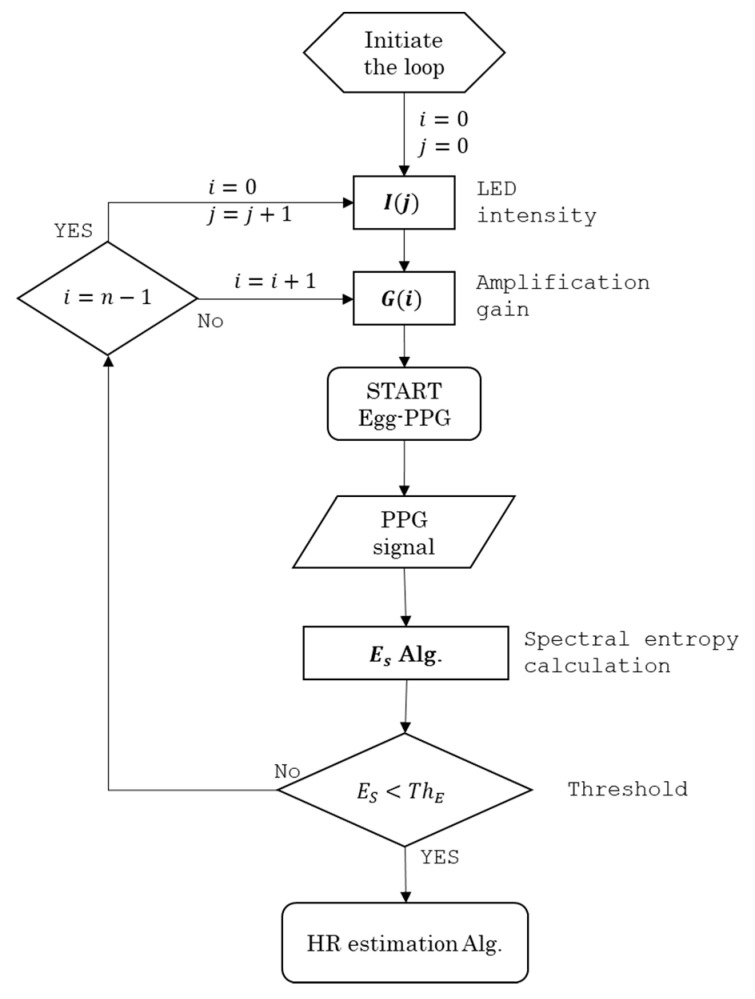
Flow chart shows the main steps of the developed cardiac signal detection algorithm based on spectral entropy ($E_{S}$).

An automatic ECW detection algorithm was developed based on normalised spectral entropy as shown in the flow chart depicted in Figure 9 and the following pseudo-code:

PPG_signal (G_i_, I_j_)		
INPUT: G_i_ ∈ {−50, −60, −70, −80, −90, −100} dB; and I_j_ ∈ {150, 200, 250, 300} mW·Sr^−1^	
LOOP initiation: i = 0 and j = 0; i ∈ {0, 1, 2, 3, 4, 5}; j ∈ {0, 1, 2, 3}
FOR: G_i_ and I_j_
    DO: record 15 s of PPG_signal (G_i_, I_j_)
    DO: calculate E_S_
IF: lower_threshold ≤ E_S_ ≤ upper_threshold
    DO: PPG_signal (G_i_, I_j_) contains ECW
    DO: continue recording
ELSE:
    DO: continue LOOP
END


The proposed algorithm is based on examining a PPG segment of 15 s at each combination of amplification gain (G) and LED intensity (I). Thus, the maximum time for the testing all combinations is 6 min, which is sufficient to detect the ECW.

**Figure 10:**
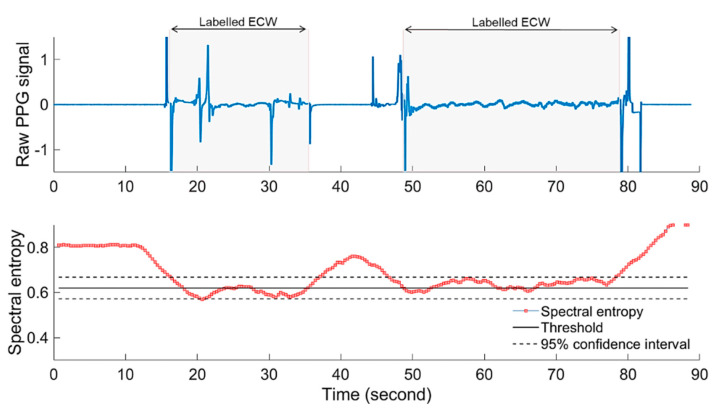
Raw PPG signal (upper graph) showing signal regions including cardiac waves (shaded regions) and regions with no cardiac waves. Additionally, the corresponding calculated spectral entropy, $E_{S}$, (lower graph) of the raw PPG signal shows the defined $E_{S}$ thresholds for the cardiac signal.

The developed ECW detection algorithm was tested offline against 10 recorded PPG segments that included cardiac (ECW) and non-cardiac epochs. The results showed that the algorithm successfully detects the ECW epochs with more than 98% accuracy. Figure 10 shows an example of a PPG segment obtained from a fertile egg at embryonic day ED09 that contains two manually labelled epochs of ECW and other non-cardiac epochs. As shown in the figure, during the ECW epochs, the calculated spectral entropy values were within the defined thresholds. It should be noted here that the developed system was validated based on visual inspection of the PPG signal, however, further validation studies are necessary to compare our system with a gold standard such as ECG [8,49].

## 5. Conclusions

In this paper, we presented a detailed design and methodology for a non-invasive PPG-based prototype (Egg-PPG) for real-time and continuous monitoring of the embryonic heart rate during incubation. A developed automatic embryonic cardiac wave (ECW) detection algorithm based on normalised spectral entropy is described. The ECW detection algorithm successfully detected the ECW epochs in the test PPG segments with 98% accuracy. The resulting heart rate of the developed embryonic heart rate estimation algorithm was compared to manually labelled and calculated heart rates. The results showed that the algorithm successfully estimated the embryonic heart rate with 98.7% accuracy. The developed prototype is a promising solution for non-invasive, real-time, and continuous monitoring of the embryonic heart rate during incubation. However, further validation work is recommended in future research.

## Abbreviations

AB	Amplification circuit board
ACG	Acoustocardiogram
APG	Acceleration Plethysmogram
BCG	Ballistocardiogram
CAM	Chorioallantoic Membrane
CWT	Continuous Wavelet Transform
DFT	Discrete Fourier Transform
DOG	Derivative of Gaussian
ECG	Electrocardiogram
ECW	Embryonic Cardiac Wave
ED	Embryonic Day
FFT	Fast Fourier Transform
HPF	High-Pass Filter
HR	Heart Rate
ICG	Impedance Cardiogram
iPPG	image Photoplethysmographic
LB	LED’s Control Circuit Board
LED	Light-Emitting Diode
LPF	Low-Pass Filter
LVR	Linear Voltage Regulator
PGA	Programmable Gain Amplifier
PPG	Photoplethysmography
SNR	Signal-to-Noise Ratio
TIA	Transimpedance Amplifier
WT	Wavelet Transform
